# Inhibition of COX-2 ameliorates murine liver schistosomiasis japonica through splenic cellular immunoregulation

**DOI:** 10.1186/s13071-022-05201-1

**Published:** 2022-04-23

**Authors:** Zhang Qi, Chen Lan, Ji Xiaofang, Tang Juanjuan, Fu Cheng, Huang Ting, Shen Erxia, Li Zi

**Affiliations:** 1 Sino‑French Hoffmann Institute, School of Basic Medical Sciences, Guangzhou Medical University, Guangzhou, 511436 Guangdong Province China; 2 Immunology Department, School of Basic Medical Sciences, Guangzhou Medical University, Guangzhou, 511436 Guangdong Province China; 3 The Second Affiliated Hospital of Guangzhou Medical University, State Key Laboratory of Respiratory Disease, Guangdong Provincial Key Laboratory of Allergy & Clinical Immunology, Guangzhou Medical University, Guangzhou, Guangdong Province China

**Keywords:** COX-2, NS398, Granulomatous inflammation, Cellular immunoregulation, *Schistosoma japonicum*

## Abstract

**Background:**

We have reported the positive association of the cyclooxygenase 2 (COX-2)/prostaglandin E2 (PGE2) axis with liver fibrosis induced by *Schistosoma japonicum* (*Sj*) infection, and TLR4 signaling controlled this axis. However, how COX-2 regulates immune response during *Sj* infection is still unclear.

**Methods:**

Hematoxylin and eosin staining was used to evaluate the effect of the COX-2-specific inhibitor NS398 on liver granulomatous inflammation and fibrosis. Flow cytometry was used to explore the frequency and amount of different immune cell infiltration in the spleen during *Sj* infection.

**Results:**

NS398 significantly reduced the size of liver granuloma, spleen, and mesenteric lymph node (MLN) and alleviated chronic granulomatous inflammation. Mechanically, this might be by decreasing the number of *Sj*-induced macrophages and T helper type 1 (Th1), Th2, T follicular helper (Tfh), T follicular regulatory (Tfr), and germinal center B (GC B) cells. There were no differences in the number of neutrophils, myeloid-derived suppressor cells, Th17 cells, regulatory T cells (Treg), or total B cells in the spleen of the mice with or without NS398 treatment.

**Conclusions:**

COX-2/PGE2 inhibition may represent a potential therapeutic approach for schistosomiasis japonica through splenic cellular immunoregulation.

**Graphical Abstract:**

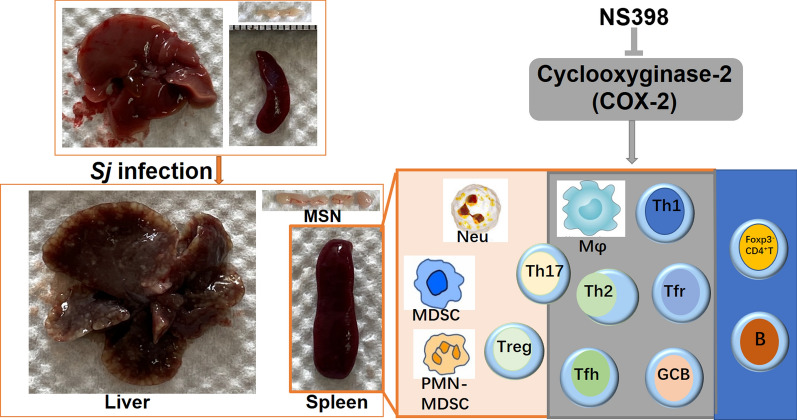

## Background

Schistosomiasis is a chronic helminthic disease affecting over 250 million people in over 78 countries [(WHO 2018), https://www.who.int/news-room/fact-sheets/detail/schistosomiasis]. The three major schistosomes infecting humans are *Schistosoma mansoni* (*Sm*), *Schistosoma haematobium* (*Sh*), and *Schistosoma japonicum* (*Sj*). *Sj* causes a hepato-intestinal form of the disease and is endemic in China, the Philippines, Indonesia, and the Mekong Delta. *Sj* reside in the mesenteric veins and hepatic portal vein, where they release eggs that induce a dramatic immune response in the intestines and liver, followed by granuloma formation, which is characterized by egg encapsulation within layers of immune cells embedded in extracellular matrix (ECM). Schistosomiasis japonica is divided into acute and chronic phases. The acute phase in murine animals occurs within the first 6 weeks after exposure, and is manifested mainly as the early liver granuloma stage [1]. In the chronic phase, hepato-intestinal or hepatosplenic disease may occur [2]. Splenomegaly is a consequence and an important clinical indicator of portal hypertension [3]. The spleen is composed of three areas—white pulp, red pulp, and a transitional zone—and serves as a filter of the blood and as one of the major peripheral immune organs. In its red pulp area, reticuloendothelial cells such as macrophages will clear away abnormal red blood cells or pathogens.

Many innate and adaptive immune cells can exert specific effects on hepatic pathology through immunoregulation. Macrophages are the most abundant in the liver granulomas of mice with *Sm* infection. Myeloid-derived suppressor cells (MDSCs), as potent suppressors of immune responses, emerge in the blood, bone marrow, or spleen during cancer, infections, or inflammation. Yang et al. found that the soluble egg Ag (SEA) and soluble worm Ag (SWA) of *Sj* enhanced the accumulation of MDSCs in the bone marrow, spleen, and mesenteric lymph nodes (MSN) [4]. The recruitment of neutrophils to the liver by IL-17A has been associated with the development of fibrosis in many chronic liver diseases including schistosomiasis japonica [5]. T lymphocytes are classified into CD4^+^ T helper (Th) cells and CD8^+^ cytotoxic T lymphocytes (CTLs). The roles of Th cells in the immune-pathogenesis of schistosomiasis have been intensely reviewed [6]. Moderate Th1 responses are included in acute schistosomiasis and early granuloma formation, while excessive Th1 response will easily lead to severe acute cachexia followed by death and is detrimental to the host. Th2 immunity acts as a double-edged sword: on the one side, it exerts anti-inflammatory effects and suppresses Th1-mediated immunopathology, but on the other side, it drives liver immunopathological damage, especially liver fibrosis. Therefore, maintaining Th1/Th2 balance is important to control the excessive pathology of schistosomiasis. Th17/IL-17 exacerbate egg-induced liver immunopathology in schistosomiasis.

T follicular helper cells (Tfh) are mainly located in the periphery of B cell follicles in secondary lymphoid organs, which regulate antigen-specific B cells to become specialized antibody producers, and aid in the formation of germinal centers (GC), affinity maturation of antibodies, somatic hypermutation and the production of memory B cells. T follicular regulatory cells (Tfr) also exist in GCs, where they play an inhibitory role in GC reactions [7, 8]. Tfh promoted liver granulomas and fibrogenesis in *Sj*-infected mice [9–11]. B cell lymphoma 6 (Bcl6)- and programmed death-1 (PD-1)-positive Tfh in the GC of murine spleen correlate with progression of liver fibrosis [10]. Tfh and Tfr increased in patients with schistosomiasis japonica [12]. Humoral immunity requires cross-talk between Tfh, Tfr, and B cells. Several studies have demonstrated that the number of B cells in the lymph nodes and spleen increases significantly during *Schistosoma* infection [13]. *Sj* recombinant fusion protein *Sj* GST-32 combined with tacrolimus (FK506) immunization augmented the induction of Tfh cells, and the expression of IL-21R on GC B cells and memory B cells increased in these immunized mice [14].

Cyclooxygenase (COX)-1 and COX-2 catalyze the first step in prostanoid biosynthesis. COX-1 is constitutively expressed whereas COX-2 is induced by certain stimuli. High COX-2 expression has been detected in several liver pathologies, while the effect of COX-2 in many liver diseases is a matter of controversy [15]. Soluble egg antigen (SEA) from *Sm* drove potent Th2 responses by triggering dendritic cells to produce COX-1, COX-2, and then prostaglandin E2 (PGE2) [16]. We have reported that COX-2 inhibitor-NS398 protected mice from hepatic fibrosis induced by *Sj* infection [17]. However, there is a lack of studies on the effects of hepatic COX-2 on immune cells during *Sj*-induced liver fibrosis.

Herein, we demonstrate the effect of NS398, a selective inhibitor of the enzymatic activity of COX-2 in PGE2 synthesis, on macrophages, neutrophils, MDSCs and Th cell subsets, B cells, and in the spleen during *Sj* infection.

## Methods

### Reagents

NS398, the inhibitor of COX-2 activity, was purchased from MedChemExpress (HY-13913, NJ, USA). Fluorescein-conjugated anti-mouse antibodies (F4/80-FITC, CD11b-PE, Gr-1-APC, Dead-APC-A750, CD45-PB450, CD45-KO525; CD4-FITC, CD3-PC5.5, IL-17A-PC7, IFN-γ-APC, IL-4-PB450; CD3-FITC, Foxp3-PC5.5, PD-1-PC7, CXCR5-APC, FVD-PB450, CD4-KO525; FAS-PE, CD19-PC5.5, B220-PC7, GL7-APC, CD138-APC-A750, FVD-PB450) and their corresponding isotype controls were obtained from eBioscience (San Diego, CA, USA). Ly6G-PC7 and Ly6G-allophycocyanin were obtained from BD Biosciences (San Jose, CA, USA).

### Mice, parasite infection, and NS398 treatment

Six- to 8-week-old female C57BL/6 mice were obtained from SPF Biotechnology Co., Ltd (Beijing) and were maintained according to institutional guidelines. All mice experiments were approved as humane by the Institutional Animal Care and Use Committee at South China Agricultural University (2019-1013). Mice were infected by 20 ± 3 *Sj* cercariae of the Chinese mainland strain through abdominal skin penetration. NS398 (3 mg/kg body weight) in 2% DMSO was administered to mice by intraperitoneal injection three times a week from week 5 to week 7 with *Sj* infection (*n* = 8), while the infection control group only received 2% DMSO (*n* = 7). Two non-infected control mice groups were treated with NS398 (*n* = 5) and 2% DMSO (*n* = 9), respectively. Mice were sacrificed at week 8 after *Sj* infection. Spleens, MSN, and liver tissues were collected for further analysis.

### H&E staining

**Fig. 1:**
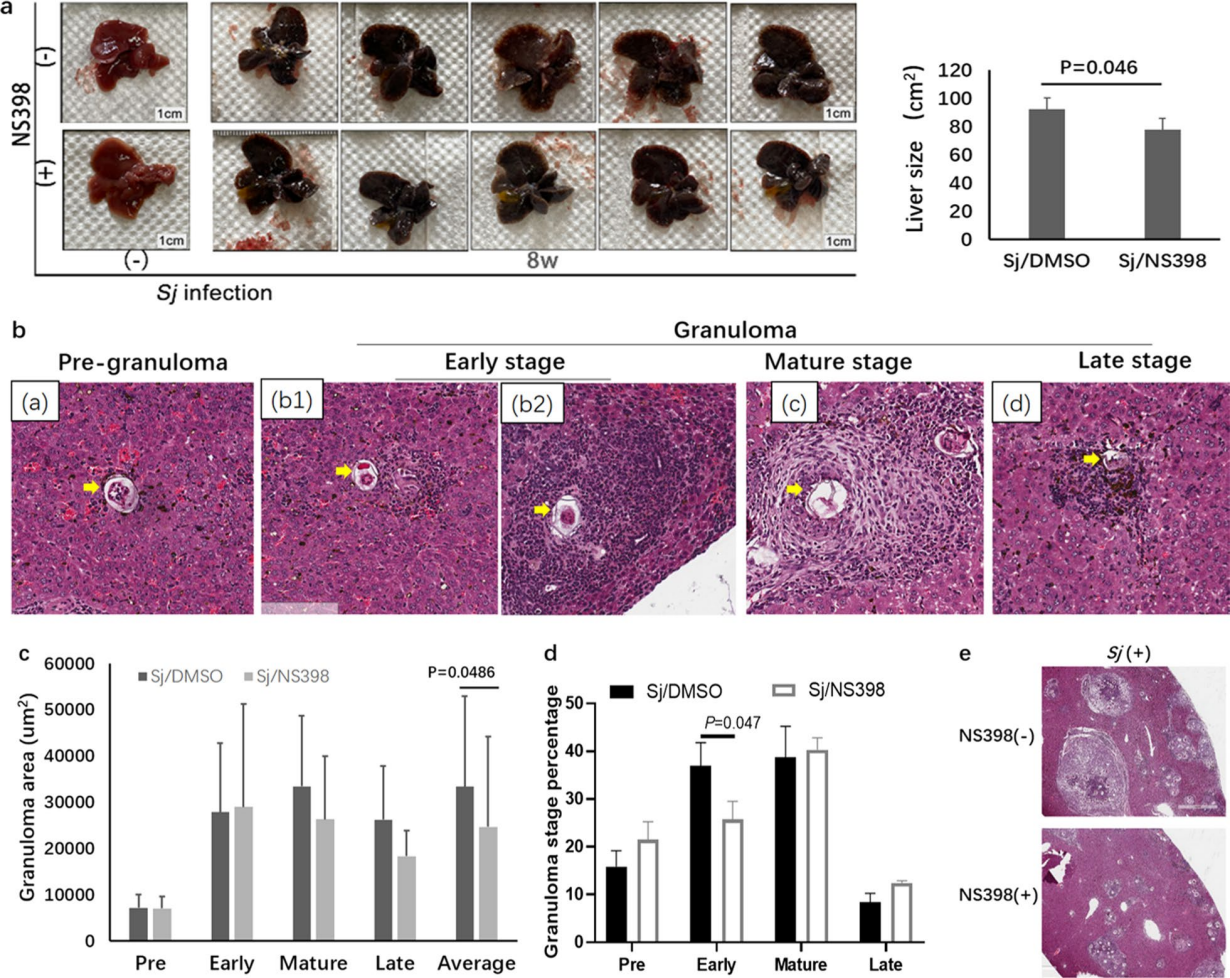
COX-2 inhibition by NS398 treatment reduced *Sj* infection-derived hepatomegaly, the size of granuloma, and the infiltration of immune cells around granuloma in the liver of mice. Each C57BL/6 mouse was infected with 20 ± 3 *Sj *cercariae. Fresh liver tissue from non-infected and infected mice with or without NS398 treatment was harvested, fixed in 4% paraformaldehyde, and embedded with paraffin, and sections were prepared for H&E staining. **a** Fresh liver tissue from indicated groups was photographed and the area (liver size) was analyzed by computer-aided design (CAD) software. **b** Typical photographs of different granuloma stages in liver sections from indicated mice groups with H&E staining, **c** the size of granuloma of each stage induced by single egg deposition, and **d** the percentage of each granuloma stage are shown. **e** Representative images from *Sj*-infected mice liver section with H&E staining without or with NS398 treatment are shown. Data in (**a** right panel) and **c**, **d** are expressed as the mean ± SD (Student’s *t*-test, statistic *P*-value is marked in the figures)

Fresh hepatic tissues were fixed in 4% paraformaldehyde for 24 h and then were embedded with paraffin. Four-micrometer liver sections were prepared and stained with hematoxylin and eosin (H&E) to assess granuloma size and the extent of liver granulomatous inflammation. The severity of liver granulomatous inflammation was evaluated according to calibrated criteria (Table 1, Fig. 1b) [1].

**Table 1:** *Schistosoma japonicum*-induced liver granuloma stages and inflammation

I. The pre-granulomatous stage—mini size, early inflammation (stage a)
a. Minimal disorganized aggregation of immune cells
b. No fibroblasts or extracellular matrix, no collagen fibers
c. Few eggs deposited
II. The granulomatous stages
(1) Early stage—small size, acute inflammation (stage b)
a. Infiltration of organized immune cells including monocytes, eosinophils, etc., around eggs
b. No fibroblasts or collagen fibers, with few or without extracellular matrix
c. Many eggs deposited
Note: Stage b1: several organized immune cell infiltration; stage b2: many organized immune cell infiltration, without or with few extracellular matrix
(2) Mature stage—big size, chronic and fibrotic inflammation (stage c)
a. Clear outer granuloma rim which is composed of a dense population of immune cells
b. Many recruited fibroblasts and their products including apparent extracellular matrix and collagen fibers inside the granuloma
c. Live or dead miracidium within the deposited eggs
(3) Late stage—small size, recovery (stage d)
Some pigmented macrophage infiltration
Disintegrated granuloma structure
Dead and calcified eggs

### Preparation of single-cell suspensions of mice spleen

Mice were anesthetized, and sterile normal saline was injected into the left ventricle to remove blood from organs. Then, the spleens were used to harvest cell suspensions by pressing these tissue pieces through a 100-mm cell strainer (BD Falcon) and then suspending in Hanks’ balanced salt solution (HBSS). Red blood cells were lysed with ammonium chloride (NH_4_Cl) for 10 min. Cell suspensions were incubated with LIVE/DEAD Zombie NIR™ Fixable Viability Kit (Biolegend) for 20 min, and then resuspended at 2–3 × 10^6^ cells/ml in complete RPMI 1640 medium with 10% fetal bovine serum (FBS).

### Cell surface staining and then flow cytometry analysis

Cell suspensions were preblocked with mouse Fc block antibody (BD, clone 2.4G2). The following antibodies were used for cell surface marker staining: anti-CD45-BV510 (clone 30-F11), anti-CD11b-PE (clone M1/70), anti-Ly6G-PE-cy7 (clone 1A8), anti-F4/80-FITC (clone BM8), anti-B220-PE-cy7 (clone RA3-6B2), anti-CD19-PerCP-Cy5.5 (clone eBio1D3), anti-PD-1-APC (clone 29F.1A12), anti-CD3e-PerCP-Cy5.5 (clone 145-2C11), anti-CD4-FITC (clone RM4-5), anti-CXCR5-Biotin (clone 2G8), anti-CD95-PE (clone 15A7) and anti-GL7-Alexa Fluor^®^ 647 (clone GL7). Flow cytometry (FACS) analysis was conducted in CytoFLEX (Beckman Coulter) and analyzed with FlowJo software (FlowJo LLC, Ashland, OR, USA).

### Intracellular cytokine staining and then flow cytometry analysis

Foxp3 intracellular staining was conducted using eBioscience™ Foxp3/transcription factor fixation/permeabilization concentrate and diluent. Cells were washed using permeabilization buffer (10×) followed by intracellular staining with anti-Foxp3-PerCP-Cy5.5 (clone FJK-1bs) for 40 min. For intracellular staining of cytokines, cell suspensions were stimulated with phorbol 12-myristate 13-acetate (20 ng/ml; Sigma-Aldrich), ionomycin (1 μg/ml; Sigma-Aldrich), and BFA (10 μg/ml; Sigma-Aldrich) for 4 h at 37 °C. Then, cells were fixed, permeabilized, and stained for anti-IL4-BV421 (clone 11B11), anti-interferon gamma (IFN-γ)-APC (clone XMG1.2), and anti-IL-17A-PE-cy7 (clone EBIO17B7). FACS was conducted and analyzed.

### Statistical analysis

The results are presented as the standard deviation (± SD) of the indicated number of replicates/experiments. Data from each group were analyzed using SPSS software (v11.0). Statistical evaluation of the difference between means was performed by one- or two-tailed, paired or unpaired Student’s *t*-test. A *P*-value of ≤ 0.05 was considered statistically significant.

## Results

### COX-2 inhibition by NS398 treatment reduced *Sj* infection-derived hepatomegaly, the size of granuloma, and the infiltration of immune cells around granuloma in the liver of mice

We have reported that the COX-2/PGE2 axis was involved in the formation of liver fibrosis induced by *Sj* infection under the control of the TLR4 pathway [17]. Chronic liver inflammation was supposed to develop into fibrosis. The size of granuloma induced by *Sj* egg deposition, especially at the mature and late stage, indicated the extent of fibrosis, and the amount of early-stage granuloma indicated the extent of inflammation. Herein, we found that COX-2 inhibition by NS398 treatment significantly attenuated hepatomegaly (Fig. 1a) (*t*-test: *Sj*/DMSO vs. *Sj*/NS398: *t*_(5)_ = 78.17, *P* = 0.046) and the average granuloma size in the liver, including the size of mature and late-stage granulomas induced by single egg deposition (Fig. 1c, e) (*t*-test: *Sj*/DMSO vs. *Sj*/NS398: *t*_(39)_ = 24,703.35, *P* = 0.0486). According to H&E staining of the liver sections, *Sj* infection-induced liver granulomas were classified into different stages (Table 1, Fig. 1b) [1]. COX-2 inhibition significantly decreased the percentage of early-stage granuloma (Fig. 1d, e) (*t*-test: *Sj*/DMSO vs. *Sj*/NS398: *t*_(5)_ = 25.74, *P* = 0.047). This suggests that NS398 alleviated the extent of both inflammation and fibrosis in the *Sj*-infected mice liver.

### NS398 decreased *Sj* infection-induced enlargement of mesenteric lymph nodes and splenomegaly of mice

**Fig. 2:**
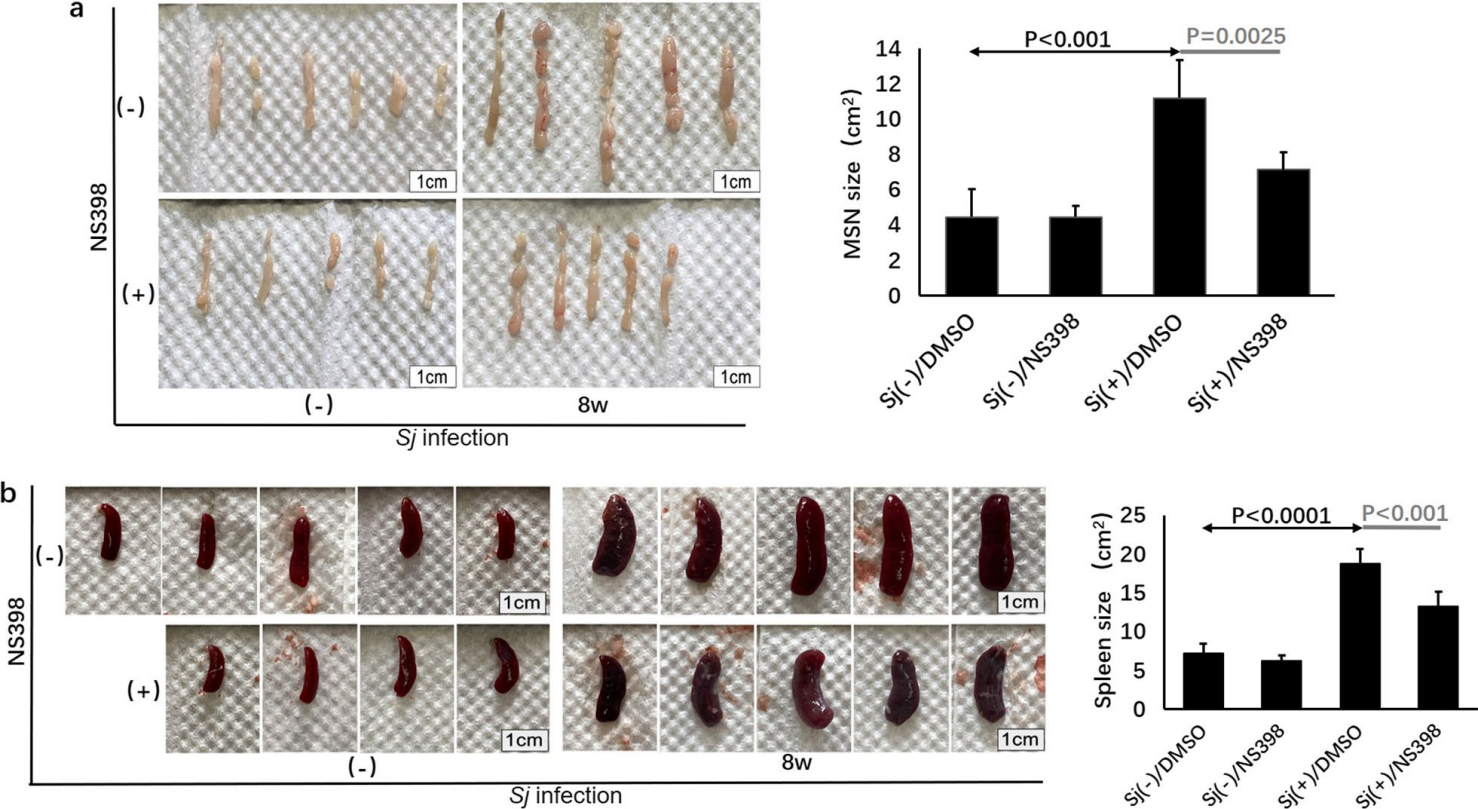
NS398 decreased *Sj* infection-induced enlargement of mesenteric lymph nodes and splenomegaly of mice. Fresh MSN (**a**) and spleen (**b**) from non-infected and infected mice with or without NS398 treatment were harvested and photographed, and the area (size) was analyzed by CAD software. Data in (**a** right panel) and (**b** right panel) are expressed as the mean ± SD (Student’s *t*-test, statistic *P*-value is marked in the figures)

Immune cells in the MLN and spleen tended to migrate or recycle into the liver and promoted hepatic inflammation and fibrosis induced by *Sj* infection [2]. The diameter of spleen thickness represents the severity of fibrosis. Herein, we showed that the size of the MLN and spleen was significantly reduced (Fig. 2) (*t*-test: *Sj*(−)/DMSO vs. *Sj*(+)/DMSO: MSN size: *t*_(5)_ = 11.18, *P* = 0.0001; spleen size: *t*_(5)_ = 18.77, *P* < 0.0001. *Sj*(+)/DMSO vs. *Sj*(+)/NS398: MSN size: *t*_(5)_ = 7.13, *P* = 0.0025; spleen size: *t*_(5)_ = 13.23, *P* = 0.0008), which suggests that NS398 decreased their contribution to liver pathogenesis during *Sj* infection.

### NS398 decreased the percentage of macrophages and did not affect the number of neutrophils or MDSCs in the spleen of *Sj*-infected mice

**Fig. 3:**
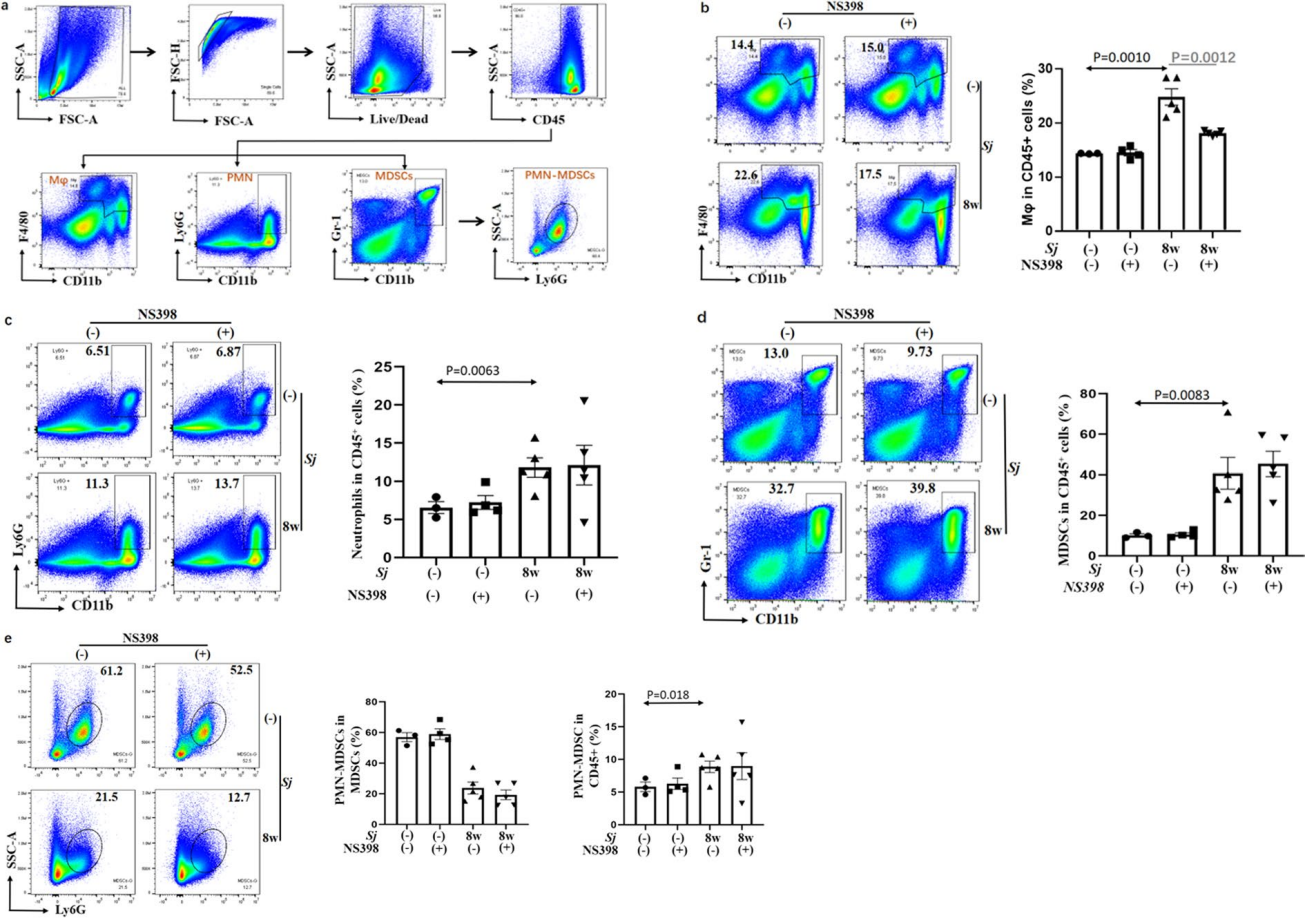
NS398 affected the number of macrophages, but not neutrophils and MDSC in the spleen of *Sj*-infected mice. Spleens from non-infected and infected mice with or without NS398 treatment were harvested and cells were isolated. **a** Spleen cells were stained with dead-APC-A750, CD45-PB450 or KO525, CD11b-PE, F4/80-FITC, Ly6G- PC7, and Gr-1-APC antibodies. F4/80 ^+^ CD11b^+^ cells (macrophage), Ly6G^+^CD11b^+^ cells (neutrophil or PMN) or Gr-1^+^ CD11b^+^ cells (MDSCs) were analyzed, and data shown are gated on CD45^+^ live cells. Ly6G^+^ cells in MDSCs (PMN-MDSCs) were also analyzed. The expression of macrophages (**b**) and neutrophils (**c**) in CD45^+^ cells in the spleen was evaluated. MDSCs in CD45^+^ cells (**d**) and PMN-MDSCs in MDSCs or in CD45^+^ cells (**e**) in the spleen were evaluated. Data are expressed as the mean ± SD of indicated numbers of mice from different groups (Student’s *t*-test, statistic *P*-value is marked in the figures)

Mice with 8 weeks of *Sj* infection will develop advanced liver fibrosis [18]. To explore the existence of macrophages, neutrophils, and MDSCs in *Sj*-infected mice spleen at week 8 of *Sj* infection with or without NS398 treatment, mononuclear cells were isolated from mouse spleen, and the percentage of CD45, CD11b, and F4/80 co-expressed macrophages (Mφ) [19], CD11b and Gr-1 co-expressed MDSCs, and CD11b and Ly6G co-expressed neutrophils were detected by FACS (Fig. 3a). Dalton et al. reported that both F4/80^high^ CD11b^low^ and F4/80^intermediate (int)^ CD11b^int^ belonged to macrophages according to their morphology during *Leishmania donovani* infection [20]. Herein, our analysis also included these macrophage subpopulations. *Sj* infection significantly increased the percentage of macrophages (*t*-test: *Sj*(−)/DMSO vs. *Sj*(+)/DMSO: *t*_(5)_ = 24.82, *P* = 0.0010), neutrophils (*t*-test: *Sj*(−)/DMSO vs. *Sj*(+)/DMSO: *t*_(5)_ = 11.80, *P* = 0.0063), and MDSCs (*t*-test: *Sj*(−)/DMSO vs. *Sj*(+)/DMSO: *t*_(5)_ = 40.74, *P* = 0.0083), especially polymorphonuclear leucocyte (PMN)-MDSCs (*t*-test: *Sj*(−)/DMSO vs. *Sj*(+)/DMSO: *t*_(5)_ = 8.86, *P* = 0.018) (Fig. 2b–e). NS398 treatment significantly lowered the percentage of macrophages (*t*-test: *Sj*/DMSO vs. *Sj*/NS398: *t*_(5)_ = 18.12, *P* = 0.0012), but it did not significantly change the percentage of neutrophils and MDSCs distributed in the murine spleen (Fig. 3b–e). Among the macrophage subpopulations, the number of macrophages with CD11b^int^F4/80^int^ was apparently increased with *Sj* infection, and decreased strikingly with NS398 treatment.

### COX-2 inhibition reduced *Sj* infection-derived Th1 and Th2 but not Th17 cells in the spleen

**Fig. 4:**
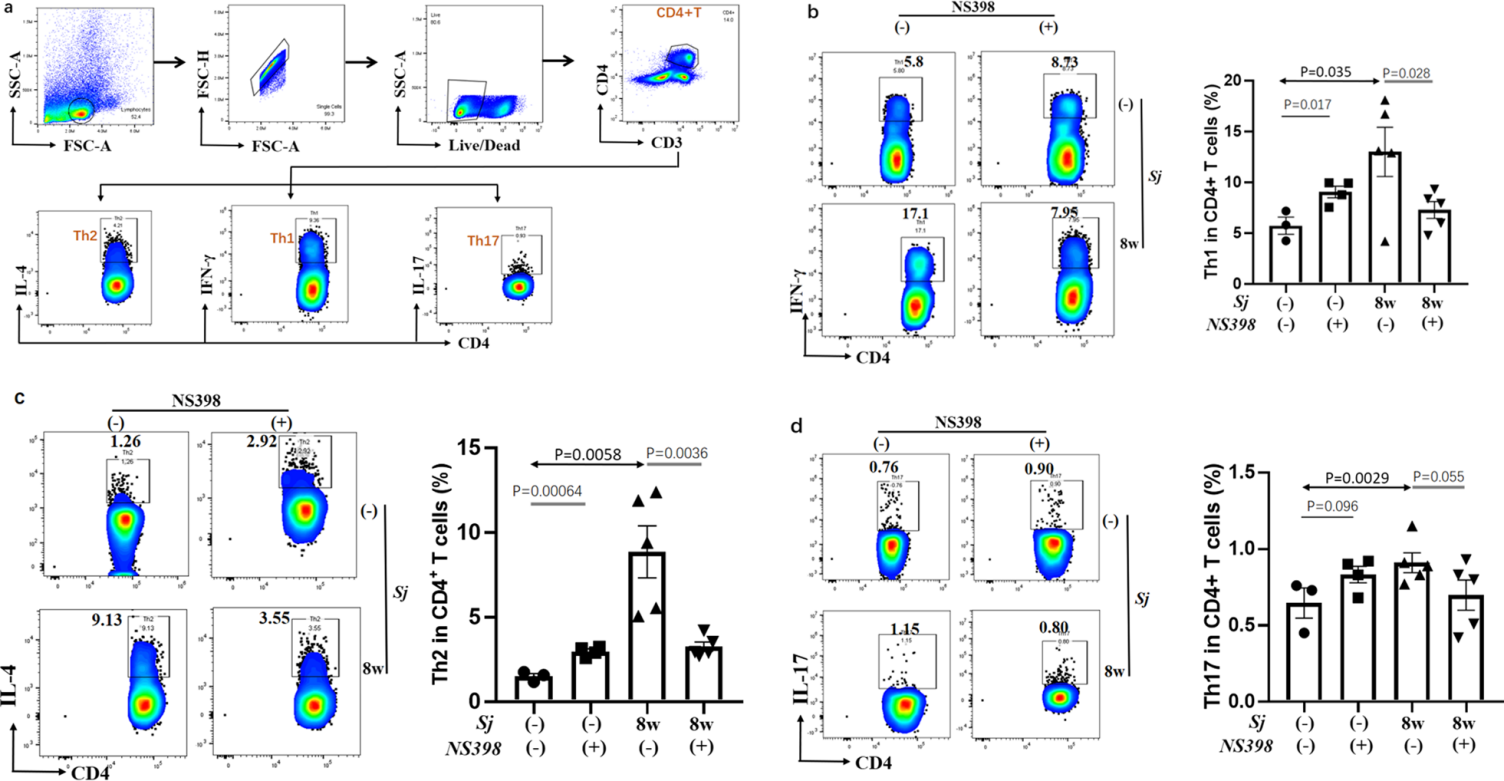
COX-2 inhibition decreased *Sj* infection-derived Th1, Th2 but not Th17 cells in the spleen. **a** Isolated spleen cells were stained with dead-APC-A750, CD3-PC5.5, CD4- FITC, IFN-γ-APC, IL-4-PB450, and IL-17-PC7 antibodies. IFN-γ^+^ CD4^+^ cells (Th1), IL-4^+^CD4^+^ cells (Th2), or IL-17^+^CD4^+^ cells (Th17) were analyzed and data shown are gated on CD3^+^CD4^+^ T cells. Numbers represent the percentage of the boxed population. **b** The expression of Th1 cells in CD3^+^CD4^+^ T cells in the spleen was evaluated. **c** Th2 or **d** Th17 cells in CD3^+^CD4^+^ T cells in spleen were evaluated. Data are expressed as the mean ± SD (Student’s *t*-test, statistic *P*-value is marked in the figures)

To investigate whether T helper cell subsets in the spleen were involved in the alleviation of mice liver pathogenesis induced by *Sj* infection during NS398 treatment, mononuclear cells from infected mouse spleen with or without NS398 treatment were stained by fluorescence-labeled anti-CD3, CD4, IFN-γ, IL-4, and IL-17A, and then were detected by FACS (Fig. 4a). *Sj* infection significantly increased the percentage of Th1, Th2, and Th17 cells (*t*-test: *Sj*(−)/DMSO vs. *Sj*(+)/DMSO: Th1: *t*_(5)_ = 13.0, *P* = 0.035; Th2: *t*_(5)_ = 8.87, *P* = 0.0058 Th17: *t*_(5)_ = 0.91, *P* = 0.029) in the spleen, and the increase in Th1 and Th2 was significantly attenuated by NS398 treatment (*t*-test: *Sj*/DMSO vs. *Sj*/NS398: Th1: *t*_(5)_ = 7.29, *P* = 0.028; Th2: *t*_(5)_ = 3.27, *P* = 0.0036) (Fig. 4b, c). However, NS398 did not affect this Th17 subpopulation in the mice spleen (*t*-test: *Sj*/DMSO vs. *Sj*/NS398: *t*_(5)_ = 0.070, *P* = 0.055) (Fig. 4d). The effect of NS398 in decreasing Th1 and Th2 differentiation explained its ability to alleviate the extent of liver granulomatous inflammation and fibrosis. In the non-infected mice with NS398 treatment, the percentage of Th1 and Th2 but not Th17 cells in the spleen was significantly increased (*t*-test: *Sj*(−)/DMSO vs. *Sj*(−)/NS398: Th1: *t*_(4)_ = 9.06, *P* = 0.017; Th2: *t*_(4)_ = 2.99, *P* = 0.00064; Th1*7: t*_(4)_ = 0.83, *P* = 0.096). However, the ratio of Th1/Th2 in non-infected mice spleen was not affected by NS398 treatment (data not shown).

### NS398 decreased *Sj* infection-triggered Tfh and Tfr cell generation

**Fig. 5:**
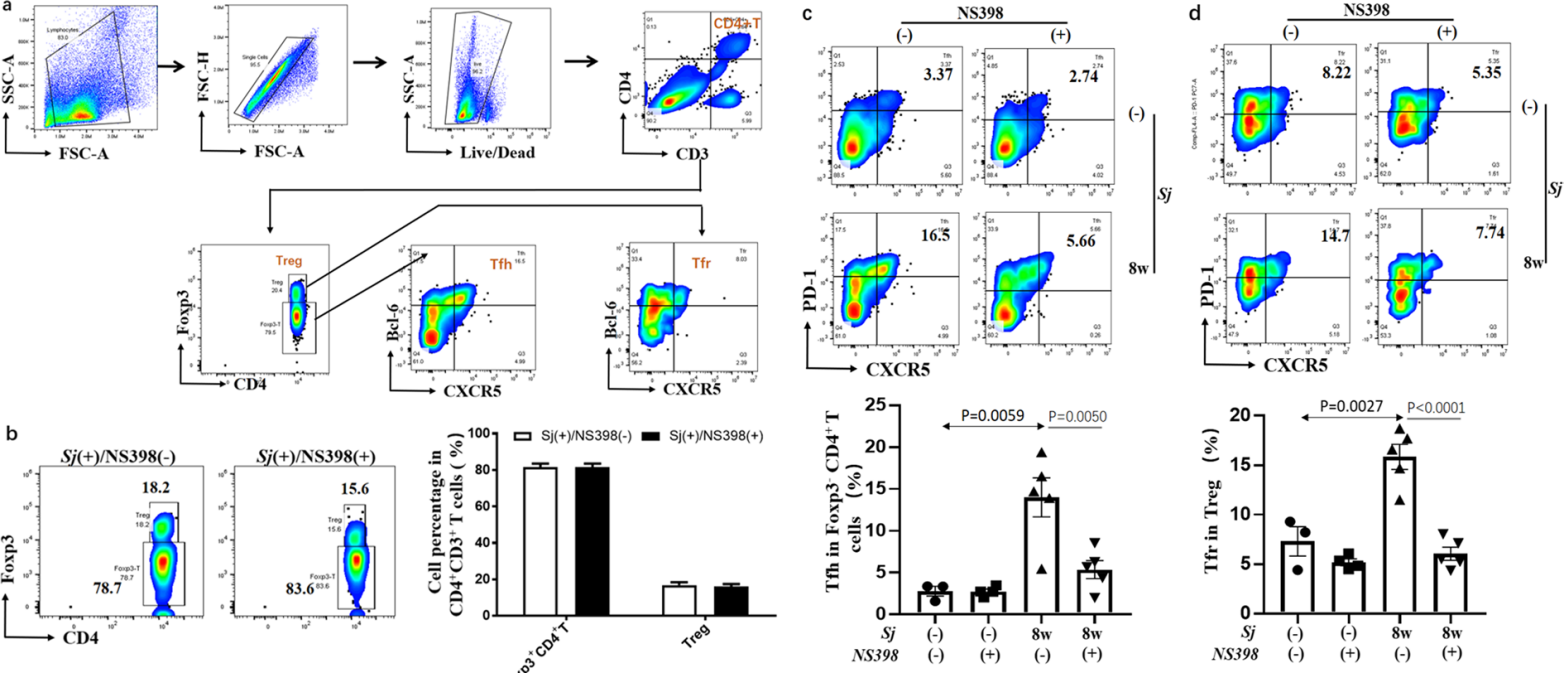
NS398 decreased *Sj* infection-triggered Tfh and Tfr. **a** Isolated spleen cells were stained with CD3-FITC, CD4-KO525, Foxp3-PC5.5, CXCR5-APC, PD-1-PC7. Foxp3^+^ or Foxp3^−^ cells were analyzed and data shown are gated on CD3^+^CD4^+^ T cells. CXCR5^+^ PD-1^+^ cells (Tfh) were analyzed, and data shown are gated on Foxp3^+^ or Foxp3^−^ CD4^+^ T cells, respectively. Numbers represent the percentage of the boxed population. **b** The expression of Foxp3^+^ or Foxp3^−^ cells in CD3^+^CD4^+^ T cells in the spleen was evaluated. **c** CXCR5^+^ PD-1^+^ cells or d CXCR5^+^ PD-1^+^ T cells (Tfr) in spleen were evaluated. Data are expressed as the mean ± SD (Student’s *t*-test, statistic *P*-value is marked in the figures)

Profoundly impaired CD4^+^ T cell responses are associated with *Sj* infection. Tfh and Tfr have been found to be increased in patients with *Sj* infection [12]. However, the function of Tfr-mediated immune responses to *Sj* infection and the effects of NS398 on this cell subset is unclear. Flow cytometry was performed to analyze Tfh and Tfr populations within spleen mononuclear cell preparations, as shown in Fig. 5a. Significantly increasing of PD-1^+^CXCR5^+^ Tfh cells (*t*-test: *Sj*(−)/DMSO vs. *Sj*(+)/DMSO: *t*_(5)_ = 13.99, *P* = 0.0059) and Tfr cells (*t*-test: *Sj*(−)/DMSO vs. *Sj*(+)/DMSO: *t*_(5)_ = 15.84, *P* = 0.0027) were shown in the *Sj*-infected murine spleen (Fig. 5c, d). Although NS398 treatment did not change the number of Foxp3^−^ and Foxp3^+^ CD4^+^ T cells (Fig. 5b), it significantly decreased Tfh cells (Fig. 5c) (*t*-test: *Sj*/DMSO vs. *Sj*/NS398: *t*_(5)_ = 5.36, *P* = 0.0050) and Tfr populations (Fig. 5d) (*t*-test: *Sj*/DMSO vs. *Sj*/NS398: *t*_(5)_ = 6.05, *P* < 0.0001). Therefore, PD-1^+^CXCR5^+^ Tfh cells and Tfr cells in the spleen might play an important role in schistosomiasis japonica, and NS398 will mitigate their effects.

### NS398 treatment reduced *Sj* infection-induced GC B cell maturation in the spleen

**Fig. 6:**
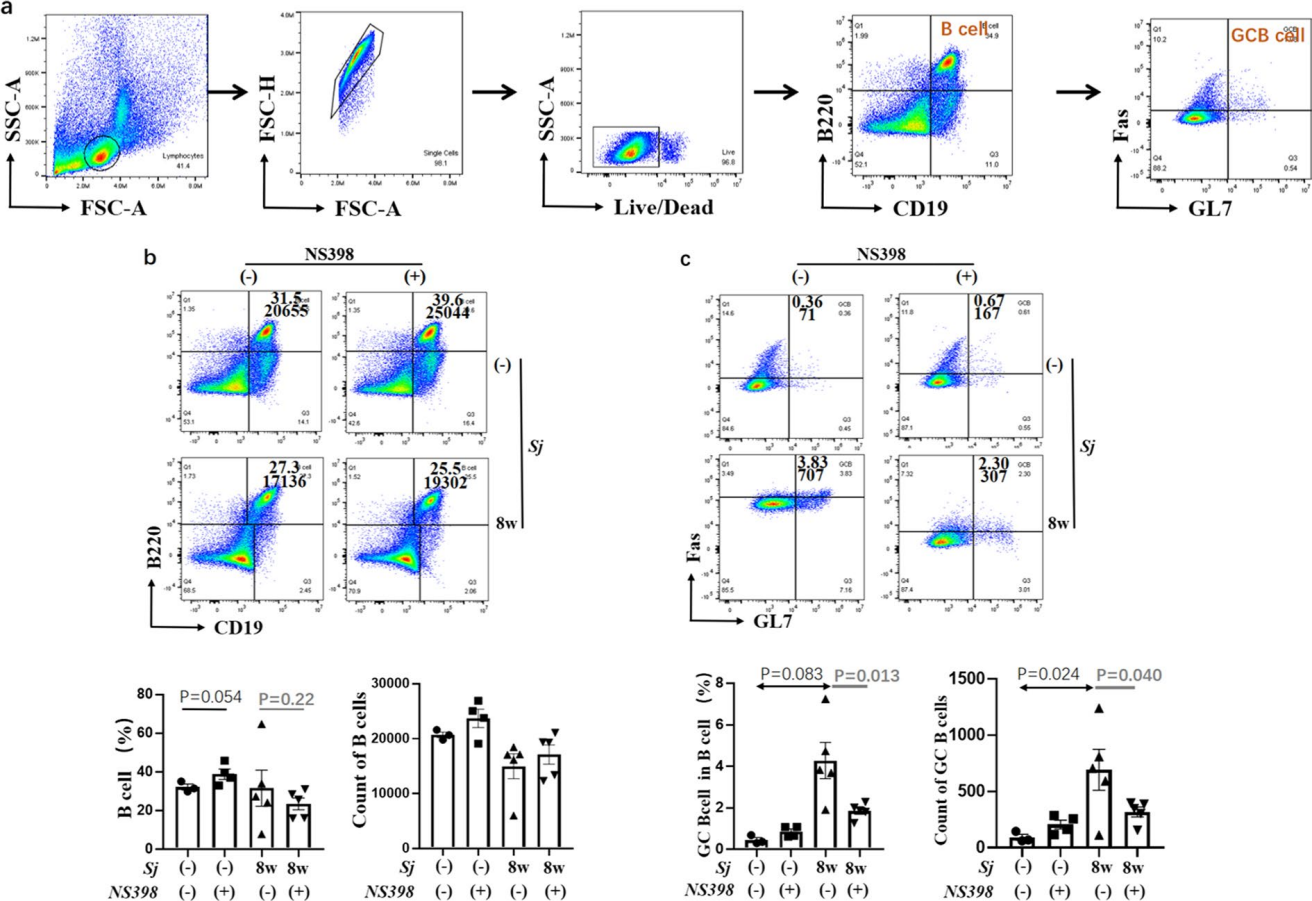
NS398 reduced *Sj* infection-induced splenic GC B cells. **a** Isolated spleen cells were stained with CD19-PC5.5, B220-PC7, Fas-PE, and GL7-APC antibodies. CD19^+^ B220^+^ cells (B cell) and GL7^+^Fas^+^ cells in B cell (GCB) were gated and analyzed. Numbers represent the percentage (up) & counts (down) of the boxed population. The expression of B cells (**b**) and GCB cells (**c**) in spleen was evaluated. Data are expressed as the mean ± SD (Student’s *t*-test, statistic *P*-value is marked in the figures)

As reported, during *Sj* infection, the number of splenic B cells was significantly increased [13, 21]. Here, we found that at week 8 of *Sj* infection, the number and percentage of B cells in the mice spleen showed no change, and GC B cells were significantly increased (*t*-test: *Sj*(−)/DMSO vs. *Sj*(+)/DMSO: percentage of GC B: *t*_(5)_ = 4.28, *P* = 0.0083; number of GC B: *t*_(5)_ = 693, *P* = 0.024) (Fig. 6). NS398 treatment showed no change in B cells, but significantly decreased the number and percentage of GC B cells (*t*-test: *Sj*(−)/DMSO vs. *Sj*(+)/DMSO: percentage: *t*_(5)_ = 1.85, *P* = 0.013; number: *t*_(5)_ = 318.8, *P* = 0.040) (Fig. 6b and c). The effect of NS398 on GC B cells was consistent with Tfh and Tfr.

## Discussion

In this study, we investigated the infiltration of multiple immune cells into the spleen of mice during 8 weeks of *Sj* infection, and the relationship between NS398 (COX-2-specific inhibitor) treatment and the number of these infiltrating immune cells and the extent of schistosomiasis japonica. We found that NS398 decreased *Sj* infection-derived hepatomegaly, the size of granuloma, and the extent of liver fibrosis and granulomatous inflammation, which was consistent with our last study [17]. The mechanisms involved might include the effect of NS398 in significantly decreasing *Sj* infection-induced enlargement of mesenteric lymph nodes and splenomegaly, and the number and percentage of macrophages, Th1, Th2, Tfh, Tfr, and GC B cells in the mice spleen. We firstly identified that 8 weeks of *Sj* infection significantly increased the number of MDSCs, Tfr, and GC B cells in the spleen. This suggested that liver pathogenesis induced by *Sj* infection might occur through the activation of the COX-2/PGE2 axis in the liver and then the induction of Th1, Th2, Tfh, Tfr differentiation, and GC B maturation in the spleen.

In terms of the effect of *Sj* infection on cellular immune regulation, Zheng et al. [22] showed that neutrophils in the spleen of C57BL/6 mice increased gradually from 6, 8, and 12 weeks of 20 cercariae infection. The relationship between neutrophils and Th17 cells was consistent in mice with schistosomiasis [22, 23], which was supported by this study. Both our results and the reported study showed that *Sj* infection significantly increased the number of macrophages in the mice spleen (CD11b^high^ or CD11b^int^ and F4/80^high^) [24]. The CD11b^low^ F4/80^int^ subpopulation was also included in macrophages in this study according to the morphological identification from the study by Dalton et al. [20]. NS398 suppressed *Trypanosoma cruzi* growth by inhibiting TGF-β production by macrophages [25]. Either recruited or resident macrophages in different tissues showed different morphological and functional phenotypes. Macrophages in the liver around or inside *Sj* egg granuloma lesions are a major cell population and an important contributor to liver fibrosis [26]. Our next study will explore the effect and mechanisms of NS398 on macrophage number, subpopulations, and activity in the liver. Yang et al. demonstrated that MDSCs in the spleen of C57BL/6 mice at 5–6 weeks of 40 ± 5 *Sj* cercariae infection were strikingly increased [4], and we showed that MDSCs in the spleen of C57BL/6 mice at 8 weeks of 20 ± 3 *Sj* cercariae infection were also significantly induced. The role and mechanisms of local and systemic MDSCs in schistosomiasis japonica need further exploration.

Huang et al. [27] showed that Th2, Th17, and CD25^+^Foxp3^+^ CD4^+^T cells (Treg) but not Th1 were significantly increased in BALB/c mice spleen at 7 weeks of 30 ± 2 *Sj* cercariae infection. Tebeje et al. [28] found that 5 weeks of 34 *Sj* cercariae-infected splenic Th1 cells responded more strongly to *Sj* adult worm antigen preparation (SWAP) compared to SEA. Th2 immune response to SEA was dominant at week 6, and Treg response was high in the CBA mice spleen at week 5 followed by a decline at week 6. Su’s group [29] identified the dynamics of Th1, Th2, Th17, and Treg cells and their role in 12 *Sj *cercariae infection in C57BL/6 mice and showed that all of these T cell subsets increased gradually in the infected mice spleen at weeks 5, 8 and 13. Elevated frequencies of Th17 cells have been shown in the *Sm*-infected C57BL/6 mice spleen at week 6, but not at week 4 or 8 [30]. In contrast to C57BL/c, CBA mice developed more severe lesions driven by Th17 cells [31]. We showed that Th1, Th2, and Th17 cells in C57BL/6 mice spleen were significantly induced by 20 ± 3 *Sj* cercariae infection for 8 weeks (Fig. 4), which is consistent with the study reported by Su’s group [29]. However, the percentage of Th17 in *Sj*-infected mice spleen was still less than 1% and was not significantly decreased by NS398 in this study. This might be explained by the relatively high dose of *Sj *cercariae infection inducing a more severe extent of liver fibrosis, and the major production of IL-17A from γδT cells in *Sj*-infected mice spleen [32]. Meanwhile, our results showed that N398 increased the number of Th1 and Th2 cells in non-infected mice spleen (Fig. 4), but the Th1/Th2 ratio showed no change compared with no NS398 treatment (data not shown). Since the homeostasis of the Th1/Th2 ratio was not affected, NS398 still possessed therapeutic potential in *Sj*-induced liver schistosomiasis. In fact, as reported, PGE2 increased Th1 differentiation in BALB/c mice and COX2 genetic deletion in C57BL/c increased Th2 differentiation [33, 34].

Tfh cells in the spleen of C57BL/6 mice at both 5 and 6 weeks of 40 ± 5 *Sj *cercariae infection were significantly increased to about 20% of Treg cells, especially at week 5 [11]. The high frequency of Tfh and Tfr cells was significantly increased in the peripheral blood mononuclear cells (PBMCs) of patients with schistosomiasis japonica [35]. Infection with *Sj* induced TGF-β- and IL-10-producing B cells while decreasing CR5^+^ B1a cells [13, 21, 36]. B cell-mediated antibody production requires cross-talk between Tfh, Tfr, and B cells [7, 8]. In our study, Tfh, Tfr, and GC B cells significantly increased to 13.99 ± 5.22% of Foxp3^−^CD4^+^T cells, 15.84 ± 2.84% of Treg cells, and 4.28 ± 1.95% of B cells, respectively, at week 8 of *Sj* infection.

COX-2 plays an important role in the progression of liver fibrosis [17, 37]. We found that the COX-2/PGE2 axis was positively associated with the extent of liver fibrosis induced by *Sj* infection [17]. Herein, we showed that NS398, the COX-2-specific inhibitor, reduced the granuloma size and ameliorated splenomegaly and the size of MSN, which supports its effect on liver inflammation and fibrosis. Macrophages in the *Sj*-infected mice spleen were significantly decreased by NS398. Macrophage deletion with clodronate significantly attenuated granuloma formation in the liver of mice induced by *Sj* infection [38]. COX-2 blockage by NS398 inhibited accumulation and function of MDSCs and promoted proliferation and inhibited apoptosis of CD4^+^ T cells in the spleen and bone marrow of mice with traumatic stress [39]. Triggering the COX-2-PGE2/EP2 pathway resulted in the induction of Th2 immune response [40]. Septic rats given NS398 showed amelioration of IL-6, tumor necrosis factor alpha (TNF-α), and CD4^+^/CD8^+^ T cell imbalance in the liver and decreased liver injury [41]. NS398 significantly increased IL-4 secretion while decreasing IFN-γ secretion by splenocytes after ovalbumin stimulation in mice with allergic skin inflammation [34]. NS398 stimulated Th1 and inhibited Th2 type cytokines which were produced by PBMCs co-cultured with supernatant of the A549 cell line [42]. However, *Staphylococcus aureus Cowan I*-induced IFN-γ production was markedly increased in spleen cells from BALB/c mice with NS398 treatment [43]. Therefore, the effect of NS398 on MDSCs, Th1, and Th2 was dependent on the disease model. Overexpression of COX-2 enhanced survival of chronic lymphocytic leukemia B cells. NS398 significantly reduced the generation of CD38^+^ IgM^+^ and CD38^+^ IgG^+^ antibody-secreting cells [44]. However, no study showed the role of NS398 in Tfh and Tfr differentiation and GC B maturation. Since both Tfh and Tfr were dominant populations in the *Sj-*-infected mice spleen and NS398 significantly decreased them, their role and mechanism in *Sj* infection-induced liver fibrosis warrant in-depth investigation.

## Conclusions

Our study firstly outlined the reciprocal relationships between the COX-2/PGE2 axis and the size of the liver, spleen, MSN and liver granuloma, and multiple immune cell infiltration in the spleen. We provided evidence that COX-2 inhibition ameliorated liver inflammation and fibrosis induced by *Sj* infection through suppression of macrophage and Th1, Th2, Tfh, Tfr and GC B cell accumulation in the spleen. COX-2/PGE2 inhibition may represent a potential therapeutic approach for schistosomiasis japonica. The detailed mechanisms of NS398 regulating these cells including through co-stimulatory molecules and their spatiotemporal regulation need further study.

## Data Availability

The datasets supporting the conclusions of this article are included within the article.

## Abbreviations

COX-2: Cyclooxygenase 2
PGE2: Prostaglandin E2
*Sj*: *Schistosoma* *japonicum*
MDSCs: Myeloid-derived suppressor cells
MLN: Mesenteric lymph nodes
Th1: T helper type 1
Tfh: T follicular helper
Tfr: T follicular regulatory cells
GCB: Germinal center B
*Sm*: *Schistosoma mansoni*
*Sh*: *Schistosoma haematobium*
ECM: Extracellular matrix
SEA: Soluble egg Ag
SWA: Soluble worm Ag
CTLs: CD8^+^ cytotoxic T lymphocytes
Bcl6: B cell lymphoma 6
PD-1: Programmed death-1
H&E: Hematoxylin and eosin
NH4Cl: Ammonium chloride
FACS: Flow cytometry
IFN-γ: Interferon gamma
SD: Standard deviation
Mφ: Macrophage
PMN: Polymorphonuclear leucocyte
Treg: Regulatory T cells
SWAP: *Sj* adult worm antigen preparation
PBMCs: Peripheral blood mononuclear cells
TNF-α: Tumor necrosis factor alpha
